# A Cardioversion and Renal Dysfunction Cascade: Cardioversion for Atrial Fibrillation, Acute Kidney Injury, and Recurrence of Atrial Fibrillation

**DOI:** 10.7759/cureus.7672

**Published:** 2020-04-14

**Authors:** Iriagbonse R Asemota, Rita Igwilo, Chineme Nwaichi, Chimezie Mbachi, Isaac Paintsil

**Affiliations:** 1 Internal Medicine, John H. Stroger Jr. Hospital of Cook County, Chicago, USA; 2 Internal Medicine, University of Nigeria, Enugu, NGA; 3 Internal Medicine, John H. Stroger, Jr. Hospital of Cook County, Chicago, USA; 4 Internal Medicine, John H. Stroger Jr Hospital of Cook County, Chicago, USA

**Keywords:** afcard, atrial fibrillation, atrial fibrillation recurrence, direct current cardioversion, dccv, renal dysfunction, aki, arrhythmia, cardio-renal cascade

## Abstract

A 62-year-old woman with hypertension presented with progressively worsening shortness of breath due to acute decompensated heart failure with atrial fibrillation in rapid ventricular response. During admission, she was managed with diuretics, goal-directed medical therapy for heart failure with successful DCCV (Direct current cardioversion) for first episode atrial fibrillation. However, one day after discharge, the patient presented with a recurrence of dyspnea with atrial fibrillation in rapid ventricular response and a reduction in urine output with elevated serum creatinine. In this case report, we describe the syndrome of acute kidney injury following cardioversion for atrial fibrillation known as AFCARD (Atrial Fibrillation Cardioversion Associated with Renal Dysfunction), highlight its incidence and reflect on renal dysfunction subserving the recurrence of atrial fibrillation after successful DCCV.

## Introduction

Renal dysfunction following direct current cardioversion (DCCV) for atrial fibrillation (AF) is a recognized complication following cardioversion for atrial fibrillation and presents with an interval worsening of renal function [1]. Post-cardioversion renal failure is heralded by renal hypoperfusion which then results in a rise in serum creatinine [2,3]. This case report illustrates the occurence of Atrial Fibrillation Cardioversion Associated with Renal Dysfunction (AFCARD), the importance of active surveillance for its occurrence in patients with atrial fibrillation following cardioversion, and the predictors of AFCARD and its effect on the recurrence of atrial fibrillation after cardioversion.

## Case presentation

A 62-year-old female with hypertension and diabetes presented to the emergency department with one month of shortness of breath, orthopnea, worsening exercise tolerance, paroxysmal nocturnal dyspnea, palpitation, and bilateral leg swelling. She had been compliant with her medications which included Nifedipine, Irbesartan and Metformin.

On admission, her vital signs were a respiratory rate (RR) of 16 cycles/min, heart rate (HR) of 102 bpm, blood pressure (BP) of 104/75 mmHg and saturating 94% on room air. Physical examination revealed bibasilar crepitations, elevated jugular venous pulsation, and bilateral pitting leg edema. Laboratory work up revealed Na-132mmol/L, K- 4.9mmol/L, Cl- 109mmol/L, HCO_3_- 27mmol/L, BUN- 17mg/dL, Cr- 1.1mg/dL, GFR- 50ml/min/1.73m2, AST-17U/L, ALT- 27U/L, HbA1c- 6.5, WBC- 4.9, HGB- 12.7, BNP-246, Troponin - normal, D-dimer- normal, TSH/T4- normal.

**Figure 1:**
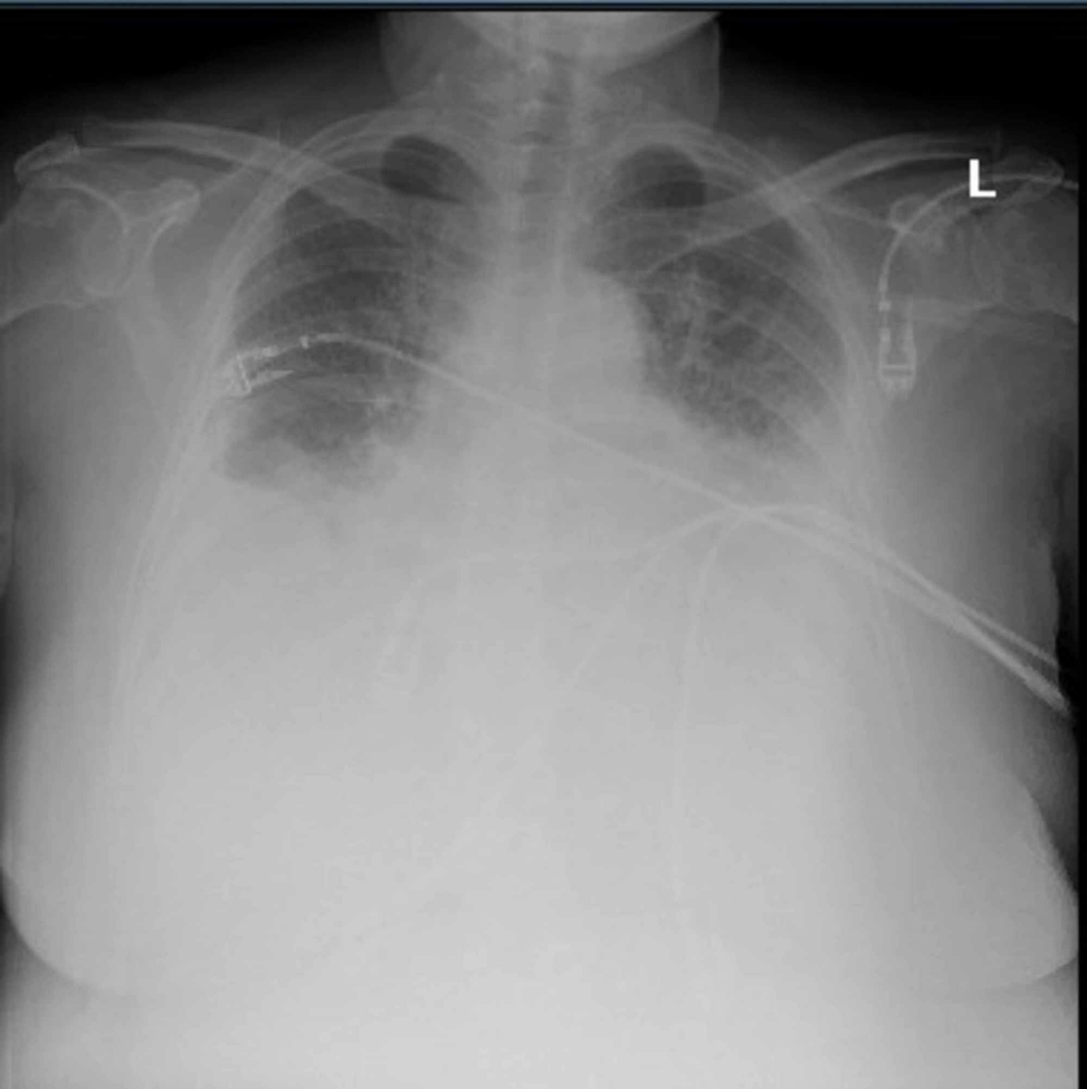
Chest xray showing small to moderate bilateral pleural effusions with associated basal consolidation/atelectasis and mild pulmonary vascular congestion suggestive of congestive heart failure.

**Figure 2:**
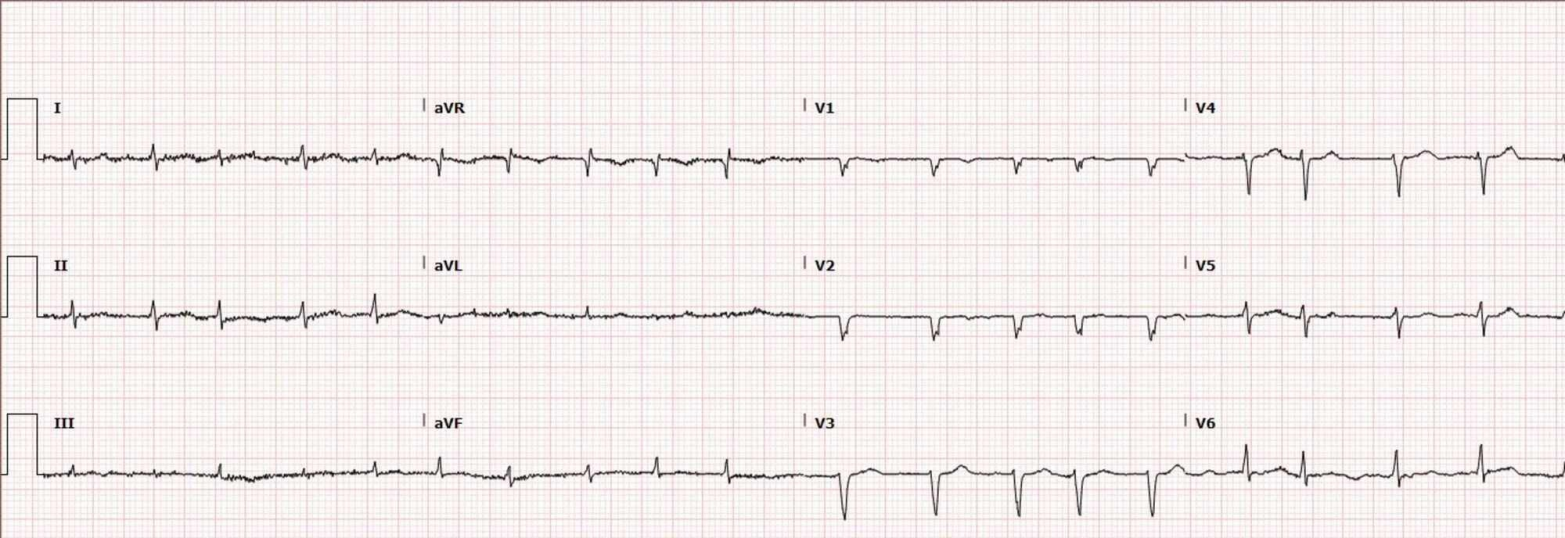
Electrocardiograph(ECG) showing Atrial Fibrillation with Rapid Ventricular Response(RVR)

**Figure 3:**
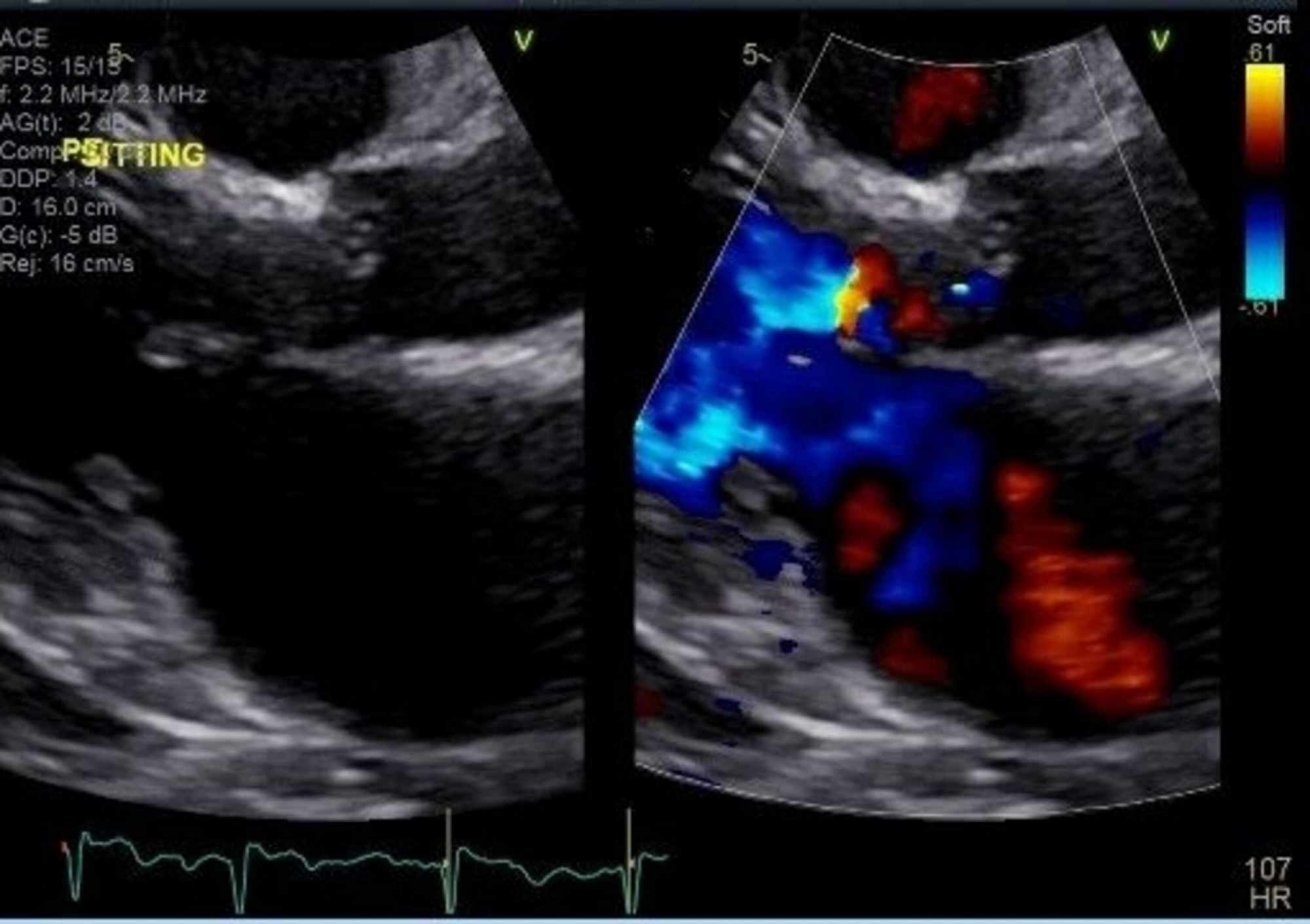
Echocardiography showing normal Left ventricular wall thickness with estimated Left ventricular Ejection Fraction of 55-65% with features consistent with pseudonormal left ventricular filling patter with concomittant abnormal relaxation and increased filling pressure (Grade 2 diastolic dysfunction). Associated aortic and mitral valve regurgitation.

Electrocardiogram revealed atrial fibrillation with the rapid ventricular rate (RVR) of 150bpm and a chest x-ray revealed bilateral pleural effusion with mild pulmonary vascular congestion. However, no pulmonary emboli were identified on computed tomography pulmonary angiogram (figures 1, 2). An Echocardiogram showed an left ventricular ejection fraction of 55-65%, with grade II diastolic dysfunction, moderate to severely dilated left atrium, severe mitral regurgitation, moderate tricuspid regurgitation (thought to be functional regurgitation, no structural valve abnormality seen), dilated inferior vena cava and increased pulmonary artery systolic pressure (figure 3).

The patient was diagnosed with acute decompensated diastolic heart failure with new-onset atrial fibrillation in rapid ventricular response with a CHADSVASc of 4. Patient was administered diltiazem initially for rate control and commenced on intravenous furosemide 40mg twice daily and later continued on metoprolol succinate PO 25 mg twice daily for rate control and Rivaroxaban 15 mg PO daily. On day 7 of admission, she had significant improvement in symptoms and was clinically euvolemic with atrial fibrillation in controlled ventricular response on metoprolol succinate. A transesophageal echocardiography (TEE) was done which showed similar findings to the initial echocardiography but with no evidence of thrombus in the atrial appendage with interval improvement in mitral and tricuspid valve regurgitation. During the index admission, we achieved a sinus rhythm with direct current cardioversion of 200 joules after the TEE and was afterward discharged on Rivaroxaban, Metoprolol succinate, Amiodarone, Losartan, and Furosemide.

However, she was admitted 24 hours after discharge with shortness of breath which got worse with exertion, orthopnea, paroxysmal nocturnal dyspnea and decreased urine output despite being compliant with her discharge medication. She was dyspneic and required BiPAP, and she was later switched to 2L intranasal oxygen by nasal cannula. Her vital signs were as follows: HR of 93bpm, RR of 45cycles/min, BP of 116/95mmHg. Examination revealed crackles at the mid lungs bilaterally and bilateral pitting edema. Laboratory investigations revealed BNP-130, BUN 56mg/dL, Cr 2.2mg/dL (initial Cr from previous admission was 1.1 mg/dL), Na- 130mmol/L, WBC- 6.2, HB 13g/dL. Urine microscopy showed many white blood cells, but no muddy casts. This admission was further complicated by bradycardia, hypotension, hyponatremia, and hyperkalemia and was managed conservatively by withholding ACEI/ARB and beta-blockers. The patient continued to receive intravenous furosemide.

A repeat transthoracic echocardiogram showed an ejection fraction of 55-65%, no wall motion abnormalities, Doppler parameters consistent with restrictive physiology indicative of decreased left ventricular diastolic compliance and/or increased left atrial pressure, right ventricular volume, and pressure overload as evidenced by the diastolic and systolic flattening of the ventricular septum, moderate mitral and tricuspid regurgitation with normal IVC size. Subsequently, the serum creatinine increased to 2.5mg/dL and then plateaued before gradually trending downwards to 1.9mg/dL after a few days (table 1). On 4th day of admission, recurrence of atrial fibrillation was noted, despite the fact that the patient was on amiodarone for rhythm maintenance after DCCV. The patient was switched to metoprolol 12.5mg and amiodarone was discontinued. The patient was seen in clinic 3 months after with a creatinine level of 1.3 showing continuing renal improvement since discharge.

**Table 1:** Daily serum creatinine levels showing interval rise in serum creatinine on day 7 after DCCV cardioversion

Day	Creatinine	BUN
1	1.2	62
2	1.3	62
3	1.3	60
4	1.4	62
5	1.4	68
6	1.3	70
7	2.2	64
8	2	62
9	2	54
10	2.2	54
11	2.5	56
13	2.3	37
14	2.3	39
15	2.4	39
16	2.4	37
17	2.2	34
18	1.9	30

## Discussion

Atrial fibrillation is the most commonly sustained arrhythmia encountered in daily practice [1]. While prospective studies have not demonstrated differences in mortality between rate control and rhythm control strategies, sinus rhythm may provide significant symptomatic improvement, and many patients with persistent atrial fibrillation undergo electrical or pharmacological cardioversion [4]. The indications for DCCV can be divided into 2 major categories: namely, the treatment of acute tachyarrhythmias and elective cardioversion for chronic atrial fibrillation and flutter. About 90% of cases of atrial fibrillation are successfully converted to sinus rhythm, whereas 95-97% of cases of ventricular tachycardia can be terminated by DCCV [5]. Another invasive procedure to restore and maintain sinus rhythm is catheter ‘ablation’ technology, but it is used less frequently than cardioversion [6].

DCCV is associated with risks including thromboembolism, complications associated with sedation/anesthesia (e.g. aspiration or respiratory arrest), sinus bradycardia, hypotension and rarely, pulmonary edema [7]. However, renal dysfunction post-DCCV (otherwise called post cardioversion renal failure) is less well described. In a recent study in 2013 evaluating the incidence and prognosis of renal dysfunction following cardioversion of atrial defibrillation, 17% of patients were reported to have developed AFCARD [1]. In another prospective study published in 2018 at the Mayo Clinic, the incidence was 5.7% [2]. In another study done in Jerusalem, post-cardioversion renal failure had an incidence rate of 9.7% [3].

Studies did not show a consensus definition of AFCARD. However, several studies alluded to AFCARD as absolute increase in serum Creatinine by ≥0.3mg/dL or a greater or equal to 50% increase compared to baseline within 48hrs of DCCV [2]. One study defined AFCARD as a rise in serum creatinine greater than 25% from baseline within a week following DCCV [1]. Another study defined post-cardioversion renal failure as a rise in serum creatinine greater than 25%, or greater than 0.5 mg/dl from baseline [3]. In the case described, the patient had been admitted and treated for heart failure secondary to new-onset AF. A week after, she underwent cardioversion and was discharged. Serum creatinine rose from the previous admission from 1.1mg/dL with eGFR of 50ml/min/1.73m2 (CKD stage 3A) to a serum creatinine of 2.2mg/dL with GFR of 23 within 48hrs of DCCV during this admission. This represented a 100% increase from baseline. A pointer to AFCARD in this patient was the timing of onset of the acute renal dysfunction in relation to cardioversion (her initial presenting symptoms had improved and urine output/renal function was normal prior to DCCV and patient was euvolemic on discharge post DCCV).

Many risk factors have been shown to be associated with AFCARD, many of which our patient had. In a study to determine the incidence, timing, risk factors and outcomes of post - cardioversion renal failure (PCRF) by Hellman et al., two strong predictors were congestive cardiac Failure and chronic renal failure [3]. Similar to the case described, a prospective maintained database review conducted over 14 years on patients undergoing DCCV at Mayo Clinic found prior diuretic use, inpatient status, and a lower heart rate post- DCCV to be strong associations [2]. The index patient in this study had similar implicating risk factors including chronic kidney disease. In addition, diabetes mellitus was seen as a predictor of AFCARD due to occult renal disease complicating renal function following DCCV [1].

In 2011, Schmidt et al. reported a series of 159 patients with persistent AF who underwent successful cardioversion in whom the renal function was assessed by estimated glomerular filtration (eGFR) at baseline and who were followed for one year in order to determine subsequent eGFR and recurrence of AF. The authors concluded that in patients with persistent AF who undergo successful cardioversion, impaired renal function is directly associated with a risk of AF recurrence [8]. Following this proven fact, it is not surprising that the patient had a recurrence of AF shortly after the post cardioversion syndrome. In one study, for example, the lower the eGFR, the more there was a likelihood of AF recurrence: eGFR <30ml/min, hazard ratio 6.82, P<0.001; eGFR 30-59ml/min, hazard ratio 3.31, P=0.01; eGFR 60-90ml/min, hazard ratio 2.10, P=0.13. In the same study, maintenance of sinus rhythm was associated with improvement in eGFR in patients with mild or moderate renal insufficiency [9].

The pathophysiology of AFCARD though not clearly understood is related to the hemodynamic changes that occur after cardioversion and attaining sinus rhythm, which results in renal hypoperfusion [1]. The outcomes of AFCARD include a higher incidence of advanced heart failure, diabetes mellitus, worsened chronic kidney disease and increased mortality rates. In the Hellman et al. study, the 1-year survival rate for patients with PCRF was 50% compared to 89% in controls [3].

There is currently an ongoing prospective study in Jerusalem, Israel, scheduled for completion in December 2020. It aims to evaluate the risk of ARF following cardioversion. Importantly, hemodynamic changes, fluid balance, and sodium levels will be evaluated as potential mechanisms for both acute renal failure and pulmonary edema post cardioversion. This holds promise as it may provide answers to the improperly understood pathophysiology of Atrial Fibrillation Cardioversion Associated with Renal Dysfunction [10]. However, DCCV resulting in renal injury is a phenomenon that needs closer attention and renal function may need to evaluated closely especially in patients with risk factors.

## Conclusions

AFCARD is a recognized phenomenon and portends bad clinical outcomes including a cascade of recurrent atrial fibrillation, heart failure, a decline in renal function and increased mortality. Therefore the anticipation of and monitoring for renal dysfunction following DCCV should be a part of post-cardioversion surveillance for patients with atrial fibrillation who achieve sinus rhythm after cardioversion.
